# Human Islet Response to Selected Type 1 Diabetes-Associated Bacteria: A Transcriptome-Based Study

**DOI:** 10.3389/fimmu.2019.02623

**Published:** 2019-11-08

**Authors:** Ahmed M. Abdellatif, Heather Jensen Smith, Robert Z. Harms, Nora E. Sarvetnick

**Affiliations:** ^1^Department of Surgery-Transplant, University of Nebraska Medical Center, Omaha, NE, United States; ^2^Mary and Dick Holland Regenerative Medicine Program, University of Nebraska Medical Center, Omaha, NE, United States; ^3^Department of Anatomy and Embryology, Faculty of Veterinary Medicine, Mansoura University, Mansoura, Egypt; ^4^Fred & Pamela Buffett Cancer Center, University of Nebraska Medical Center, Omaha, NE, United States; ^5^Eppley Institute for Research in Cancer, University of Nebraska Medical Center, Omaha, NE, United States

**Keywords:** autoimmunity, *Bacteroides dorei*, β-cell, dysbiosis, *Ruminococcus gnavus*, type 1 diabetes

## Abstract

Type 1 diabetes (T1D) is a chronic autoimmune disease that results from destruction of pancreatic β-cells. T1D subjects were recently shown to harbor distinct intestinal microbiome profiles. Based on these findings, the role of gut bacteria in T1D is being intensively investigated. The mechanism connecting intestinal microbial homeostasis with the development of T1D is unknown. Specific gut bacteria such as *Bacteroides dorei* (BD) and *Ruminococcus gnavus* (RG) show markedly increased abundance prior to the development of autoimmunity. One hypothesis is that these bacteria might traverse the damaged gut barrier, and their constituents elicit a response from human islets that causes metabolic abnormalities and inflammation. We have tested this hypothesis by exposing human islets to BD and RG *in vitro*, after which RNA-Seq analysis was performed. The bacteria altered expression of many islet genes. The commonly upregulated genes by these bacteria were cytokines, chemokines and enzymes, suggesting a significant effect of gut bacteria on islet antimicrobial and biosynthetic pathways. Additionally, each bacteria displayed a unique set of differentially expressed genes (DEGs). Ingenuity pathway analysis of DEGs revealed that top activated pathways and diseases included TREM1 signaling and inflammatory response, illustrating the ability of bacteria to induce islet inflammation. The increased levels of selected factors were confirmed using immunoblotting and ELISA methods. Our data demonstrate that islets produce a complex anti-bacterial response. The response includes both symbiotic and pathogenic aspects. Both oxidative damage and leukocyte recruitment factors were prominent, which could induce beta cell damage and subsequent autoimmunity.

## Highlights

- Gut dysbiosis and impaired gut permeability are features of type 1 diabetes (T1D).
- *Bacteroides dorei* (BD), *Ruminococcus gnavus* (RG) are two bacteria increased at time of onset of T1D.
- Exposure of isolated human islets to BD or RG revealed bacteria-specific alterations in gene expression profiles.
- The genes upregulated by both BD and RG were dominated by cytokines and chemokines suggesting an islet antibacterial response involving subsequent recruitment of immune cells.
- Findings of our study uncovered key features of the islet response toward invading bacteria.

## Introduction

Type 1 diabetes (T1D) is a life-threatening disease with a rapidly increasing incidence in children and adolescents (1). T1D is thought to result from the destruction of insulin producing pancreatic β-cells by self-reactive T cells that infiltrate the islets (2). However, its etiology remains poorly understood. The genetic susceptibility to T1D is associated with specific human leukocytes antigen (HLA) alleles (3), though <10% of genetically susceptible people develop clinical T1D (4). This variation demonstrates that non-genetic factors, e.g., those contributed by the environment, could play an important role in pathogenesis of the disease (5).

The gastrointestinal tract (GIT) represents the largest compartment in the body where the host experiences the external environment (6). Intestinal biopsies from children with T1D revealed distinctive inflammatory changes (7). Moreover, increased expression of inflammatory cytokines in the duodenal mucosa of T1D patients was associated with altered composition of the gut microbiome (dysbiosis) (8). In this regard, several recent studies have also reported intestinal dysbiosis in individuals with newly diagnosed T1D (9–13). Differential abundance of certain microbial communities e.g., increased *Bacteroides* and decreased short chain fatty acids (SCFA)-producing bacteria, was reported as a common feature in T1D by the aforementioned studies. *Bacteroides dorei* and *Ruminococcus gnavus* are two bacterial species noted to be increased in infants genetically susceptible to T1D at/before development of autoantibodies (14, 15). An increased intestinal permeability was also reported in children at risk for T1D (16). The disruption of the gut epithelial barrier and subsequent leakage of microbial products into pancreas could be a predisposing factor to T1D, as suggested by Korsgren et al. (17). Indeed, whether bacterial products can elicit a response within the pancreas remains largely unknown. Costa et al. (18) noted translocation of gut bacteria to pancreatic lymph nodes (PLNs) of streptozotocin (STZ)-injected mice, an experimental model of T1D, by both morphological and molecular approaches. Such translocation was thought to contribute to the pathogenesis of T1D by activating pathogenic T helper 1 (Th1) and Th17 cells that expanded in the PLNs and pancreas. Despite the many advances in our knowledge of changes in the gut microbiome during T1D progression, their role in pathogenesis remains unknown. The intrinsic response of human islets to specific bacteria, especially those of the gut in T1D subjects remains unknown.

In the present study, we experimentally tested the hypothesis that intestinal dysbiosis and leakage of bacteria into the human pancreas could trigger an anti-bacterial immune response from islets. To achieve that aim, isolated human islets were exposed to *Bacteroides dorei* or *Ruminococcus gnavus*, two bacteria overrepresented in the gut just before/at incidence of autoimmunity in T1D susceptible individuals (14, 15) and *Escherichia coli*, a ubiquitous bacterium associated with accelerated maturation of the gut microbiome (19, 20) for 6 or 24 h. Islets exposed to the potent cytokine IL-1β for the same time periods were used as positive controls. To gain insights into the mechanisms associated with the islet specific antibacterial response, next generation sequencing (NGS) was performed. Differentially expressed genes (DEGs) were subjected to core analysis using Ingenuity Pathway Analysis (IPA) software. IPA analysis revealed distinct sets of transcripts, canonical pathways, upstream regulators, diseases, and networks specific to each bacterium. The list of DEGs common to all bacteria and IL-1β at both 6 and 24 h are dominated by cytokines, chemokines, and enzymes. Our results identify unique bacteria-specific transcriptional changes revealing both protective and pathogenic features of the islet bactericidal response.

## Materials and Methods

### Bacterial Culture and Preparation of Inocula

*Bacteroides dorei* (BD, Leibniz Institute DSMZ-German Collection of Microorganisms and Cell Cultures Cat# 17855), *Escherichia coli* (EC, Invitrogen Cat# 18258012), and *Ruminococcus gnavus* (RG, ATCC, Cat# 29149) were purchased from commercial vendors and grown according to standard protocols. Both BD and RG were grown anaerobically in tryptic soy broth supplemented with Oxyrase (Oxyrase Inc.) and resazurin salt (Sigma-Aldrich) while EC was grown aerobically in Luria-Bertani broth. All bacteria were allowed to reach early/mid-stationary phase of growth. At the end of culture, bacteria were fixed in 4% paraformaldehyde (PFA) for 30 min, rinsed three times with sterile PBS and then reconstituted in sterile physiological saline. To ensure all bacteria were killed, bacterial stocks were used to inoculate additional broth. Absence of bacterial growth 48 h after inoculation under standard culturing conditions indicated all bacteria were killed by the PFA exposure. For confirmation of the purity of tested bacteria, genomic sequencing was performed for each bacterial strain (Supplementary Table 1).

### Study Subjects

Primary human cadaveric islets from eight non-diabetic donors (21–58 years old) were ethically obtained from Prodo Laboratories Inc (Aliso Viejo, CA, USA). The donor characteristics are provided in Supplementary Table 2.

### Culture and Viability Assessment of Islets

**Figure 1:**
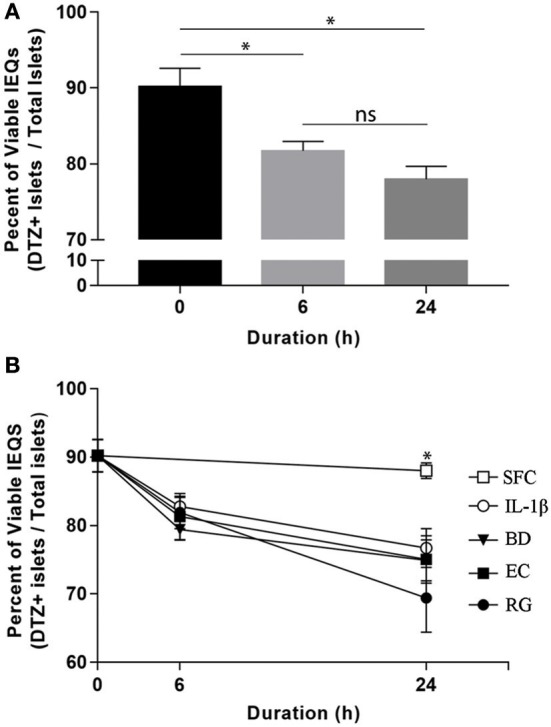
Optimized bacterial and IL-1β exposures induced significant and comparable reductions in human islet viability. **(A)** Aggregate viable IEQs across all treatment conditions significantly decreased (~20%) from the initial (0 h) to the experimental end points. **(B)** No significant differences in viable IEQs were observed in serum free controls (SFC) or between individual bacteria (BD, *B. dorei*; EC, *E. coli*; RG, *R. gnavus*) exposure groups while bacteria-exposed islets displayed reduced viabilities relative to SFC at 24 h, indicating significant, but comparable reductions in viability across bacteria. Data are shown as averages ± standard error of the mean. ^*^P < 0.05. Ns, not significant.

Upon arrival, islets were initially incubated for 6–24 h at 37°C in CMRL-1066 medium (Gibco, USA) supplemented with 2 mM l-glutamine, 1% penicillin-streptomycin, and 10% fetal bovine serum (FBS). After that, individual donor islets were cultured in 4-well plates containing FBS-free media at 1,000 islet equivalents (IEQs) per well. Each well was exposed to one of the three bacterial strains at 10 bacteria per cell (BpC) for 6 or 24 h. Time matched controls were also included using IL-1β (InvivoGen) at 1 ng/ml (positive control) and FBS-free media (negative control). Bacteria and IL-1β concentrations were considered appropriate if they induced significant changes in the percent of viable IEQs in a given treatment within 24 h. In order to monitor the viability of IEQs throughout different incubations, a dithizone (DTZ) assay was performed according to the standard protocol from the National Institutes of Health (NIH) at the beginning and end of each experiment (21). Islet viability was 90.2 ± 2.4% at the onset of the exposure period and declined, on average, <20% throughout the treatment period (Figure 1A). While there was no significant difference between the experimental conditions (Figure 1B), the percentage of viable IEQs significantly decreased from the zero point to 6 h and from the 6 to 24 h. This limited, yet significant, reduction in islet viability was essential for detecting the changes in transcript levels in the remaining islets. At the end of exposure, ~500 IEQs from each condition were prepared for RNA isolation while ~300 IEQs were prepared for protein extraction and subsequent western blot analysis. Supernatants were additionally collected from each sample. All samples were subsequently flash frozen on dry ice and stored at −80°C until further use.

### RNA Sequencing

A total of 42 samples [serum-free control (SFC), IL-1β-6 h, and EC-6 h = 6 each and IL-1β-24 h, EC-24 h, BD-6 h, BD-24 h, RG-6 h & RG-24 = 4 each] derived from six human islet donors exposed to various treatment conditions were analyzed using NGS. Briefly, total RNA was isolated using Qiagen RNeasy Minikits (Qiagen) according to the manufacturer's recommendations. Next, RNA integrity and concentration were assessed using an Agilent Bioanalyzer 2100 (Agilent) and Nanodrop Spectrophotometer, respectively. After that, cDNA libraries were generated using a SciClone NGS Library Production Robot (Perkin Elmer) and library quality was verified using the Agilent Bioanalyzer. RNA sequencing (RNA-Seq) was subsequently performed using an Illumina HiSeq2500 system (Illumina, San Diego, CA, USA) to achieve single 100 base pair reads with a read depths of 15–20 million reads per sample. Short raw sequence reads were downloaded from the HiSeq2500 server in FASTQ format and individually mapped to the human reference genome (hg38) using TopHat. The complete bioinformatics analysis pipeline for the RNAseq data was performed using the Tuxedo protocol, which includes TopHat, Cufflinks, Cuffmerge, and Cuffdiff (22). Differential gene expression analysis results from the Cufflinks analysis of individual samples were reported as Fragments Per Kilobase of transcript per Million Mapped reads (FPKM) which reports the normalized expression values for a given gene and accounts for multiple reads for an individual fragment. Transcripts up- and downregulated relative to controls were considered significantly changed only when the false discovery rate (FDR) adjusted *p*-value (*q*-value) was <0.05. Relative differences in gene expression were assessed by comparing the log2-fold change values between the islet FPKMs for bacteria-treated and time-matched controls. For easier interpretation of obtained datasets, all log2-fold change values were converted to their equivalent numeric values. Fastq files and RNA-Seq processed data were deposited into the NCBI GEO repository under the accession number GSE131320.

### Ingenuity Pathway Analysis and Data Visualization

The obtained datasets of DEGs were filtered using cut-offs of ± 2 and 0.05 for fold change and *q*-value, respectively. The generated lists of DEGs from all treatments were uploaded into the IPA software (Ingenuity Systems, Redwood City, CA) for core analyses. Initially, each group was analyzed separately using default parameters for altered canonical pathways, upstream regulators, diseases, and networks. After that, all groups were compared with each other to elucidate both shared and unique features induced by each treatment. *Z*-score is a statistical measure of the match between expected relationship direction and observed gene expression (23). Changes with an activation *z*-score ≥2 or ≤ −2 were considered significant. To show canonical pathways with highest significant changes, the list of differentially expressed pathways were further trimmed at *p*-value ≤0.05 in the IPA software. Venn diagrams of shared and unique DEGs were done using an online tool from Bioinformatics & Evolutionary Genomics (24). Heat maps of DEGs common to all groups at 6 and 24 h were generated using ClustVis (25).

### Protein Extraction and Immunoblotting

Following culture, pancreatic islets were immediately rinsed with ice-cold PBS followed by lysis with Mammalian-Protein Extraction Reagent (M-PER, Pierce Biotechnology, MA, USA) supplemented with Halt Protease and Phosphatase Inhibitor cocktail (Thermo Scientific). The concentrations of the extracted proteins were then determined with Bio-Rad total protein assay (Bio-Rad, USA). Protein lysates were diluted in 4X Laemmli buffer, heated at 95°C for 5 min, and two micrograms of the extracted proteins were loaded per each lane onto a 4–15% precast polyacrylamide gel (Bio-Rad, USA) for electrophoresis and electro-transferred to polyvinylidene difluoride (PVDF) membranes (Thermo Scientific). The membranes were then blocked in 5% non-fat dry milk in TBS-0.1% Tween-20 (TBST) for 2 h at room temperature. Blocked membranes were then incubated overnight at 4°C with primary antibodies against NAMPT (dilution 1:2,000, P4D5AT, Enzo Life Sciences Inc., NY, USA), TRAF3IP2 (1:200, sc-100647, Santa Cruz Biotechnology Inc., CA, USA), SOD2 (1:10,000, #13141, Cell Signaling Technology MA, USA), and GAPDH (1:10,000, sc-365062, Santa Cruz Biotechnology). The next day the membranes were washed three times in TBST and incubated for 1 h at room temperature with corresponding peroxidase-conjugated secondary antibodies (Jackson ImmunoResearch Laboratories, PA, USA) at dilution rates of 1:40,000 for NAMPT and 1:80,000 for others. Bound antibodies were visualized using ECL (SuperSignal West Femto Maximum Sensitivity Substrate or SuperSignal West Pico PLUS Chemiluminescent Substrate (Pierce Biotechnology) depending on the expected signal strength) and recorded on X-ray films. Immunoblots were stripped and reprobed with GAPDH antibody, served as loading control, and the relative band intensities were measured using the ImageJ software 1.46r (National Institutes of Health; http://imagej.nih.gov/ij/).

### Enzyme Linked Immunosorbent Assay

Sandwich ELISA kits from R&D Systems (R&D Systems Inc., Minneapolis, USA) for IL-8 (Cat# DY208-05), CXCL1 (Cat# DY275-05), CCL20 (Cat# DM3A00), and CXCL6 (Cat# DGC00) were used for measuring their concentrations in islet culture supernatants per manufacturer instructions. All samples were assayed in duplicate.

### Statistical Analysis

Graphs for western blot and ELISA were drawn using GraphPad Prism 7 (GraphPad Software, CA, USA). The differences between groups were determined using the ANOVA test. *Post-hoc t*-tests were run to evaluate group-specific differences when warranted by a significant ANOVA. *P*-values ≤0.05 were considered significant.

## Results

### Bacteria and IL-1β Induce Differential Gene Expression in Isolated Human Islets

**Figure 2:**
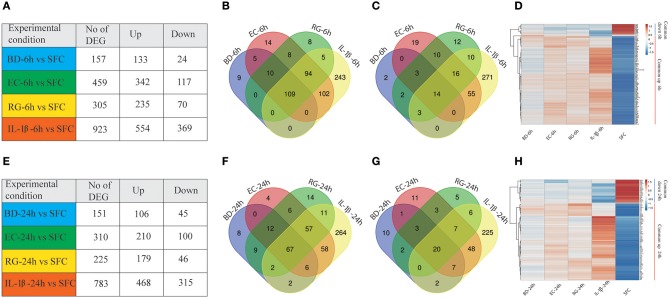
Differentially expressed genes (DEGs) at 6 and 24 h by all groups. **(A,E)** Number of DEGs in human islets after individual bacteria or IL-1β exposure for 6 or 24 h, respectively. **(B,C)** Venn diagrams indicating the overlap of up- **(B)** and down-regulated **(C)** genes at 6 h in all groups. **(D)** Heatmap of the hierarchical clustering of the shared DEGs at 6 h depicting their expression patterns. **(F,G)** Venn diagrams indicating the overlap of up- **(F)** and down-regulated **(G)** genes at 24 h in all groups. **(H)** Heatmap of the hierarchical clustering of the shared DEGs at 24 h depicting their expression patterns. Genes were clustered based on log2 of their expression values using Pearson's correlation and complete linkage function. The color key indicates the direction of changes: red and blue depict significant up- and down-regulation, respectively.

Gene expression profiling of human islets incubated with BD, EC, RG, or IL-1β revealed alterations in gene expression levels at both 6 and 24 h (Figure 2, Supplementary Table 3). Among the three tested bacteria, EC was associated with the highest number of DEGs at 6 and 24 h (459, 310), followed by RG (305, 225), and BD (157, 151). IL-1β treatment altered the expression of 923 and 783 genes at 6 and 24 h, respectively. These DEGs were dominated by up-regulated transcripts common to all bacterial treatments at both 6 and 24 h (Figures 2A,E).

Transcripts with shared and unique expression patterns were found among the different experimental conditions at each time point (Figures 2A–C,E–G). Different numbers of DEGs shared by two or more treatments were also seen and changed with increased bacteria exposure time. For instance, five upregulated genes are uniquely shared by BD and EC at 6 h, but none at 24 h. Conversely, no upregulated genes were exclusively shared by BD and RG at 6 h, though nine genes specific to both groups are found at 24 h. Heatmaps of DEGs common to all groups at 6 and 24 h are plotted in Figures 2D,H, respectively.

### Canonical Pathways Modulated by Bacteria and IL-1β in Human Islets

**Figure 3:**
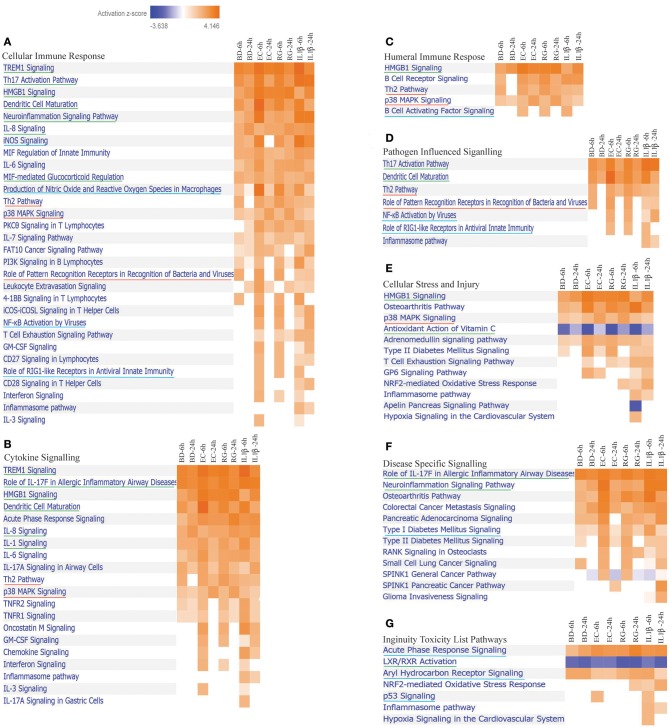
Differentially expressed canonical pathways in human islets by all treatments at 6 and 24 h. Heatmaps indicating activation *z*-scores for differentially expressed canonical pathways; **(A)** Cellular Immune Response, **(B)** Cytokine Signaling, **(C)** Humeral Immune Response, **(D)** Pathogen Influenced Signaling, **(E)** Cellular Stress and Injury, **(F)** Disease Specific Signaling, and **(G)** Ingenuity Toxicity List Pathways. The color key indicates the direction of changes: orange color shows predicted overall activation of the pathway and blue color indicates predicted overall inhibition of the pathway. Only pathways with absolute *z*-score ≥ 2 or ≤ −2 and *p*-value ≤0.05 (in the IPA software) are shown. Pathways common to all treatments (underlined in green), pathways predominantly regulated by bacteria (underlined in red), and pathways shared only by some treatments (underlined in blue) are illustrated.

IPA is a web-based application for analysis and interpretation of data derived from different omics experiments, such as RNA Sequencing and microarrays. It provides a powerful tool to uncover the significance of data and identify new targets or candidate biomarkers within the context of biological systems. IPA core analysis is an advanced platform that allows for pathway analysis, networking data, upstream effect analysis, disease relationships, and functional analysis. Lists of DEGs by all groups were submitted separately to IPA software for core analysis. After that, all groups were compared using comparison analysis. Islet treatment with bacteria and IL-1β revealed modulation of 83 canonical pathways [*z*-score ≥2 (for activation) and ≤ −2 (for inhibition) and *p*-value ≤0.05 in the IPA software] (Supplementary Table 4). From this list, we focused on those pathways related to the following categories of Ingenuity Canonical Pathways: Cellular Immune Response, Cytokine Signaling, Pathogen Influenced Signaling, Cellular stress and Injury, Disease Specific Signaling and Toxicity List Pathways (Figure 3). At the set threshold, eight signaling pathways were commonly activated by all treatments at both 6 and 24 h (*underlined in green in* Figure 3). These pathways are Neuroinflammation, IL-1, HMGB1, Dendritic Cell Maturation, TREM1, Role of IL-17F in Allergic Inflammatory Airway Diseases, MIF-mediated Glucocorticoid Regulation, and Th17 Activation. On the other hand, Role of Pattern Recognition Receptors in Recognition of Bacteria and Viruses, Th2 signaling, and p38 MAPK signaling pathways are highly activated by bacteria, but not by IL-1β. Noteworthy, there was a preferential activation of specific canonical pathways by either BD, RG, or EC at 6 h (Supplementary Table 4). BD specifically upregulated tRNA Charging, while RG exclusively activated NGF Signaling and SPINK1 General Cancer Pathway. EC revealed a preferential remarkable activation of Interferon Signaling, IL-3 Signaling and Leukocyte Extravasation Signaling. Such preferential pathway activation by certain treatments reflects the unique ability of the islets to respond to distinct bacterial stimuli. It is also noteworthy that T1D Signaling and Role of RIG1-like Receptors in Antiviral Innate Immunity are clearly upregulated at 6 h by EC, RG, and IL-1β, but not by BD.

Antioxidant action of Vitamin C is the only pathway inhibited/downregulated at 6 h by all treatments. Inhibition of the nuclear receptor LXR/RXR activation was evident in all groups with more pronounced inhibition by BD and IL-Iβ at 24 h and RG at both 6 and 24 h.

### Upstream Regulators, Diseases and Gene Network Induced Bacteria, and IL-1β in Human Islets

IPA core analysis including upstream effect, diseases, and networks analyses were performed for all DEGs. The top predicted upstream regulators, diseases, and *in silico* generated networks are summarized in Table 1. Upstream effect analysis is usually used to identify upstream factors that might drive the expression of DEGs. Similarities were observed between bacteria and IL-1β treated groups. Lipopolysaccharide (LPS) and TNF are two upstream regulators shared by all groups at both 6 and 24 h. In addition, IL-1β is common to all bacterial treatments at one or both time points. It is noteworthy that the poly rI:rC-RNA, an anti-viral response factor, was predicted to be uniquely activated by BD at 6 h.

**Table 1:** Summary of top upstream regulators, diseases and disorders, and networks associated with exposure to bacteria or IL-1β at 6 and 24 h.

**Experimental condition**	**Top upstream regulators**			**Top diseases and disorders**			**Top networks**		
	**Name**	***z*-score**	***p*-value**	**Name**	***p*-value**	**No of focus molecules**	**Name**	**Score**	**No of focus molecules**
BD-6 h vs. SFC	LPS	8.4	4.27E-54	Cell death and survival	8.26E-32	51	Hematological disease, immunological disease, organismal injury and abnormalities	30	17
	TNF	7.4	7.2E-49	Inflammatory response	1.03E-31	63			
	poly rI:rC-RNA	6.7	1.02E-47	Cellular movement, immune cell trafficking	2.63E-29	58			
BD-24 h vs. SFC	IL1β	6.9	1.79E-48	Dermatological diseases and conditions	5.51E-33–9.61E-09	57	Cell-mediated immune response, cellular development, cellular function and maintenance	25	15
	LPS	7.4	3.59E-44	Organismal injury and abnormalities	5.51E-33–3.22E-08	148			
	TNF	7.7	1.23E-43	Inflammatory response	4.84E-31–3.28E-08	86			
EC-6 h vs. SFC	TNF	11.7	6.04E-101	Inflammatory response	7.83E-58–8E-14	248	Inflammatory disease, cellular growth and proliferation, lymphoid tissue structure and development	36	24
	IL1β	9.9	1.63E-90	Organismal injury and abnormalities	7.83E-58–7.65E-14	438			
	LPS	11.3	2E-89	Cellular movement	2.77E-54–6.05E-14	202			
EC-24 h vs. SFC	IL1β	8.3	9.38E-66	inflammatory response	6.66E-41–1.8E-09	160	Cellular development, cellular growth and proliferation, hematological system development and function	35	22
	TNF	9.6	1.06E-63	Organismal injury and abnormalities	3.03E-40–2.15E-09	300			
	LPS	8.8	1.04E-61	Dermatological diseases and conditions	6.31E-40–2.12E-09	89			
RG-6 h vs. SFC	TNF	9.8	1.72E-71	Inflammatory response	1.35E-48–1.7E-11	163	Humoral immune response, protein synthesis, cell-to-cell signaling and interaction	31	20
	NFκB	8.7	1.33E-69	Organismal injury and abnormalities	2.77E-44–2.39E-11	292			
	LPS	10.4	2.27E-68	Hematological system development and function	7.56E-44–2.55E-11	147			
RG-24 h vs. SFC	IL1β	8.1	1.55E-63	Inflammatory response	1.21E-41–5.64E-10	121	Cellular development, cellular growth and proliferation, connective tissue development and function	29	18
	TNF	9.0	2.75E-58	Organismal injury and abnormalities	2.59E-36–6.11E-10	217			
	LPS	9.2	7.14E-56	Hematological system development and function	9.1E-36–5.91E-10	113			
IL1β-6 h vs. SFC	IL1β	10.4	1.22E-97	Inflammatory response	1.64E-49–1.24E-13	331	Cell-to-cell signaling and interaction, immunological disease, inflammatory disease	37	28
	TNF	13	1.17E-87	Cellular movement	2.3E-49–1.25E-13	330			
	LPS	11.8	1.77E-80	Immune cell trafficking	4.55E-48–1.25E-13	222			
IL1β-24 h vs. SFC	IL1β	9.5	3.4E-79	Inflammatory response	8.05E-48–4.06E-11	312	Cell morphology, cellular development, cellular growth and proliferation	37	27
	TNF	10.7	2.4E-75	Organismal injury and abnormalities	1.5E-47–4.43E-11	737			
	NFκB	8.3	1.79E-66	Cellular movement	6.19E-43–3.75E-11	290			

Regarding diseases and disorders associated with the datasets of DEGs, inflammatory response and organismal injury and abnormalities are shared by all treatments, though with different numbers of focus molecules. For instance, the Islet Inflammatory Response to EC was associated with activation of 248 and 160 molecules at 6 and 24 h, respectively. On the other hand, BD Islet Inflammatory Response was associated with only 63 and 86 molecules at 6 and 24 h, respectively. RG treatment was associated with 163 and 121 molecules at 6 and 24 h, respectively. Such variation in number of genes associated with each bacteria represents a scaling of the islet response toward the different stimuli based on their unique molecular constituents.

### DEGs Common to All Treatments and Those Unique to Each Bacteria

Due to similarities in the activation of important canonical pathways, upstream regulators and disease associations among different bacterial exposures, we next focused on DEGs commonly expressed by each treatment at the two studied time points. This latter set of molecules could help uncover the intrinsic islet responses against invading bacteria and/or their products. First, we analyzed genes co-regulated at both 6 and 24 h by each treatment, then compared subsequent lists of intersected genes.

**Figure 4:**
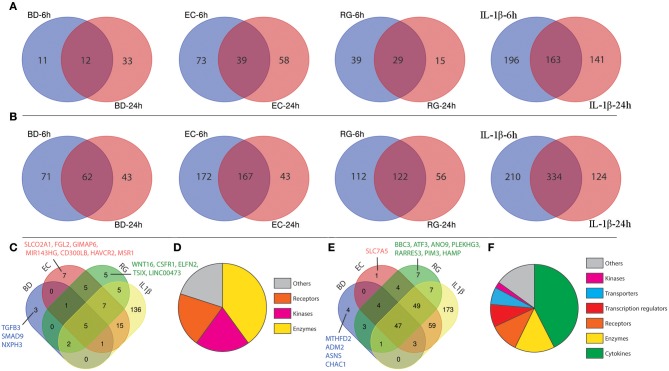
Shared differentially expressed genes (DEGs) specific to each tested bacterium after 6 and 24 h. **(A)** Venn diagrams indicating the overlap of down-regulated genes at both 6 and 24 h in each group. **(B)** Venn diagrams indicating the overlap of up-regulated genes at both 6 and 24 h in each group. **(C)** Intersection of down-regulated genes shared by each treatment. **(D)** Functional annotations of genes commonly down-regulated in all groups. **(E)** Intersection of up-regulated genes shared by each treatment. **(F)** Functional annotations of genes commonly up-regulated in all groups.

From the lists of downregulated genes, BD, EC, RG, and IL-1β downregulated the expression of 12, 39, 29, and 163 genes at both 6 and 24 h (Figure 4A, Supplementary Table 5). Further intersection of these genes revealed five genes universally downregulated by all bacteria, as well as, IL-1β. These genes are mainly enzymes, kinases, and receptors (Figures 4C,D, Table 2, and Supplementary Table 6) such as *EPHA4, MS4A6A, PRSS2, OXGR1*, and *PLCE1*.

**Table 2:** Differentially expressed genes (DEGs) shared by all treatments at both 6 and 24 h.

**Symbol**	**BD-6 h**	**BD-24 h**	**EC-6 h**	**EC-24 h**	**RG-6 h**	**RG-24 h**	**IL1β-6 h**	**IL1β-24 h**
**CYTOKINES/CHEMOKINES**								
IL6	6.1	5.6	17.5	13.3	11.6	8.4	60.7	46.7
**CCL20**	3.1	4.4	14.9	10.1	7.8	5.8	43.0	26.5
**CXCL1**	4.0	4.8	12.2	10.0	8.8	6.8	38.9	22.6
CXCL5	4.5	6.9	13.2	11.0	7.2	6.7	36.6	33.0
CSF2	6.2	7.7	14.4	5.8	10.9	7.4	35.2	4.4
**CXCL8**	5.8	6.6	14.0	12.3	11.1	9.2	33.7	20.1
IL7	4.8	3.1	10.7	6.5	11.1	6.3	30.6	13.6
CXCL3	3.5	4.2	9.5	6.2	7.1	4.7	29.7	12.4
CXCL2	3.7	4.2	8.3	7.5	8.2	5.8	28.6	13.4
**CXCL6**	2.5	4.1	7.3	8.9	5.2	5.2	20.4	29.7
IL1β	19.4	14.4	27.9	15.7	24.5	16.8	18.3	10.6
LIF	3.1	2.4	6.3	4.6	4.8	3.2	17.7	7.9
IL1A	4.5	3.6	12.6	6.0	6.4	3.9	15.1	5.4
**NAMPT**	3.2	2.9	5.3	4.6	4.6	3.8	9.7	8.6
IL23A	9.3	3.2	12.5	3.3	12.5	4.6	7.5	4.1
CSF1	2.4	2.0	4.4	2.9	3.8	2.9	7.1	4.3
TNFSF15	2.4	2.7	3.6	2.5	3.2	2.5	6.9	3.1
CCL2	2.2	2.1	3.5	2.2	3.0	2.0	4.6	3.0
CCL4	11.0	5.9	10.9	5.2	14.6	6.5	4.2	3.6
CCL3	15.4	12.9	17.6	10.2	20.5	14.6	4.2	4.9
**ENZYMES**								
ZC3H12A	2.7	3.0	5.0	5.3	5.5	4.6	15.6	11.1
**SOD2**	3.2	3.4	7.1	7.2	6.3	6.3	15.0	15.8
PTGS2	2.9	3.1	5.5	6.1	4.5	6.5	9.2	11.6
BIRC3	3.0	2.8	4.8	3.8	5.2	3.7	8.9	4.6
TNFAIP3	3.8	3.2	5.3	3.7	5.1	4.1	5.8	2.9
PLA2G4C	2.7	2.7	3.6	2.8	3.1	3.1	3.3	2.2
PLCE1	−2.3	−2.9	−3.9	−3.6	−2.6	−2.5	−6.3	−6.9
MMP1	2.2	2.4	3.0	2.8	2.2	2.4	3.9	3.9
PRSS2	−3.0	−2.6	−4.3	−4.2	−2.7	−2.9	−4.3	−2.7
**RECEPTORS**								
FFAR2	3.4	2.7	5.1	3.6	7.3	4.6	11.5	5.6
PTAFR	2.1	2.4	5.3	3.3	3.2	2.2	5.9	3.6
OXGR1	−2.4	−2.1	−2.9	−2.6	−2.9	−2.9	−5.2	−5.5
ICAM1	5.0	3.1	8.2	4.5	8.6	4.5	10.8	5.5
IL17RB	4.1	4.4	5.4	5.9	5.1	7.7	5.4	6.7
IL7R	3.0	2.2	5.9	3.2	4.5	2.2	5.3	4.3
**KINASES**								
IRAK2	2.6	2.2	3.6	2.4	3.5	2.5	4.1	3.0
EPHA4	−2.3	−2.2	−2.7	−2.4	−2.8	−2.2	−2.7	−3.1
**TRANSCRIPTIONAL REGULATORS**								
NFκBIA	4.6	3.4	6.5	4.5	7.0	4.9	10.6	6.2
CEBPD	2.4	2.2	3.9	3.8	4.2	3.2	8.1	6.5
NFκB2	6.1	3.1	7.1	3.3	7.3	3.7	7.3	3.6
RELB	4.9	2.8	5.3	2.9	5.6	3.3	4.5	2.4
**TRANSPORTERS**								
SLC6A14	2.6	3.4	9.0	4.7	4.5	2.6	17.0	13.7
SLC30A2	2.8	3.4	3.3	4.6	4.6	7.0	6.3	8.9
SLC7A11	2.9	2.5	3.1	3.0	2.3	2.2	2.3	3.5
**OTHERS**								
FGB	2.5	4.8	6.8	7.7	4.2	6.5	13.5	17.3
SNPH	2.5	2.8	4.9	6.2	6.2	7.0	12.1	9.9
SOCS1	3.3	2.6	6.7	5.3	5.4	3.7	11.9	11.3
TNFAIP2	2.4	2.1	4.5	2.6	3.7	2.1	6.5	2.7
NCCRP1	4.2	3.3	6.2	4.9	3.8	3.7	6.4	6.1
GRASP	2.2	2.1	2.8	2.5	2.9	3.1	3.0	3.1
NEAT1	2.1	2.1	2.1	2.4	2.5	2.5	3.0	3.0
MS4A6A	−4.7	−5.4	−6.8	−8.6	−6.3	−4.9	−3.3	−3.4

*The color key indicates the direction of changes: red and green colors depict the fold changes of significant up- and down-regulated genes, respectively. Factors selected for validation of RNA-Seq data using western blotting or ELISA are highlighted in bold*.

In a similar way, from the lists of upregulated genes, BD, EC, RG, and IL-1β upregulated the expression of 62, 167, 122, and 334 genes at both 6 and 24 h (Figure 4B, Supplementary Table 5). Further intersection of these genes revealed 47 genes universally upregulated by all bacteria as well as IL-1β. These 47 genes are categorized as follows: 42.6% cytokines, 14.9% enzymes, 10.6% receptors, 8.5% transcription regulators, 6.4% transporters, 2% kinases, and 14.9% others (Figures 4E,F, Table 2, and Supplementary Table 6). Commonly upregulated cytokines and chemokines include several interleukins e.g., *IL-1A, IL-1B, IL-6, IL-7*, and *IL-23A*; members of C-C motif chemokines e.g., *CCL2, CCL3, CCL4, and CCL20*; members of C-X-C motif chemokines e.g., *CXCL1, CXCL2, CXCL6, and IL-8* and *NAMPT*. Upregulated enzymes are as follows: *ZC3H12A, SOD2, PTGS2, BIRC3, TNFAIP3*, and *PLA2G4C*. Upregulated receptors are *FFAR2* (free fatty acid receptor 2), *PTAFR, ICAM1, IL17RB*, and *IL7R*. Transcription regulators are *CEBPD, NFKBIA*, and *RELB*. Upregulated transporters are *SLC30A2, SLC6A14*, and *SLC7A11*. *IRAK2* (Interleukin 1 receptor associated kinase 2) is the only receptor kinase shared by all three bacteria and IL-1β.

This analysis also revealed genes uniquely up- and downregulated by each bacterium (Figures 4C,E). In this regard, BD uniquely upregulated four genes (*MTHFD2, ADM2, ASNS, CHAC1*) and downregulated three genes (*SMAD9, NXPH3, TGFB3*). EC uniquely upregulated only one gene (*SLC7A5*) and downregulated seven genes (*SLCO2A1, FGL2, GIMAP6, MIR143HG, CD300LB, HAVCR2, MSR1*). Finally, RG uniquely upregulated seven genes (*ANO9, PLEKHG3, BBC3, ATF3, RARRES3, PIM3, HAMP*) and downregulated five genes (*LINC00473, TSIX, CSF1R, ELFN2, WNT16*).

Next, we measured the protein expression of six factors from the commonly upregulated genes using either western blotting (islet lysates) or ELISAs (islet culture supernatants). Lysates from one donor were immunoblotted using specific antibodies against NAMPT, SOD2, and TRAF3-Interacting Protein 2 (TRAF3IP2, also called ACT1). *NAMPT* and *SOD2* are from the list of commonly upregulated factors (fold change ≥ 2). *TRAF3IP2* is an NF-κB activator commonly upregulated by all treatments at a lower cut off (fold change ≥1.5). Additionally, protein concentrations of four cytokines, within the list of commonly upregulated genes, namely CCL20, CXCL1, CXCL6, and IL-8 were measured in islet culture supernatant.

**Figure 5:**
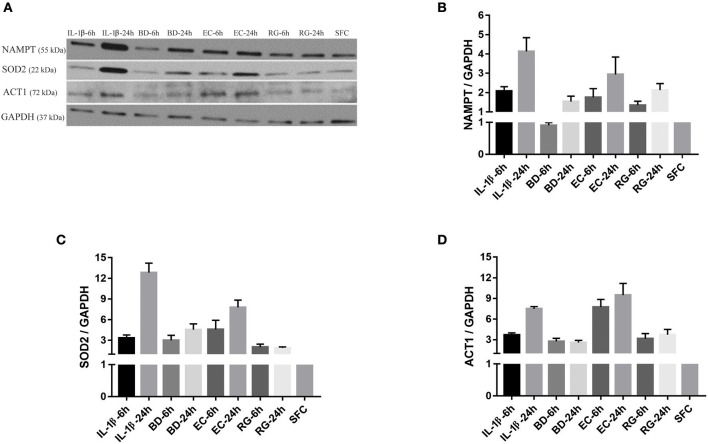
Protein expression levels for NAMPT, SOD2, and ACT1 was increased after islet incubation with bacteria and IL-1β. **(A)** Representative immunoblots for three upregulated factors of interest; NAMPT, SOD2, and ACT1 from islet lysate of donor seven. **(B–D)** Except for NAMPT at 6 h treatment with BD, the three factors revealed an overall increase in their expression at all-time points, compared to control islets. Data are shown as averages of fold changes relative to controls ± standard deviations and are representative of three independent experiments.

*NAMPT* mRNA fold changes, as indicated by RNA-Seq, were 3.2 (6 h BD), 2.9 (24 h BD), 5.3 (6 h EC), 4.6 (24 h EC), 4.6 (6 h RG), 3.8 (24 h RG), 9.7 (6 h IL-1β), and 8.6 (24 h IL-1β). With the exception of 6 h BD, a similar increase, though at lower magnitude, in NAMPT protein expression was noted (Figures 5A,B). *SOD2* mRNA fold changes were 3.2 (6 h BD), 3.4 (24 h BD), 7.1 (6 h EC), 7.2 (24 h EC), 6.3 (6 h RG), 6.3 (24 h RG), 15 (6 h IL-1β), and 15.8 (24 h IL-1β). A corresponding increase in all groups, though at lower magnitude than that observed for RNA levels, in SOD2 protein expression was observed by western blotting (Figures 5A,C). ACT1 mRNA fold changes were 1.6 (for BD), 2.0 (6 h EC), 1.6 (24 h EC), 2.2 (6 h RG), 1.8 (24 h RG), 2.5 (6 h IL-1β), and 1.8 (24 h IL-1β). A corresponding increase, but surprisingly at a higher magnitude, in ACT1 protein fold changes was detected (Figures 5A,D).

**Figure 6:**
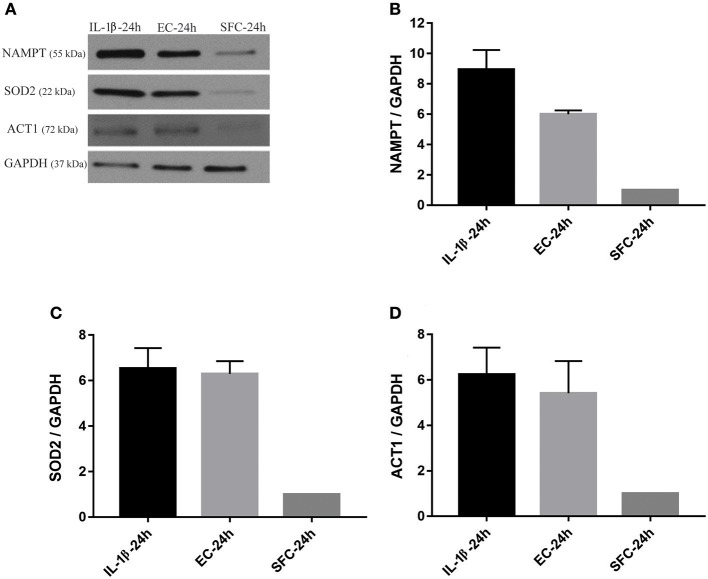
Increased protein expression levels for NAMPT, SOD2, and ACT1 in islets exposed to bacteria and IL-1β were maintained during subsequent hyperglycemia. **(A)** Representative immunoblots for NAMPT, SOD2, and ACT1 from islet lysate of donors 3 and 4 challenged with 16 mM glucose for 4 h following initial incubation with IL-1β or EC for 24 h. **(B–D)** The three factors revealed an overall increase in their expression in both treatments relative to control islets. Data are shown as averages of fold changes relative to controls ± standard deviations and are representative of three independent experiments.

The increase in the three aforementioned proteins was also tested using biological replicates. Islets from another two donors not used for RNA-Seq were initially treated with IL-1β and EC for 24 h and subsequently further stressed with hyperglycemia. At the end of exposure to bacteria or IL-1β, islets from IL-1β, EC, and SFC were stimulated with 16 mM glucose for 4 h. As shown in Figures 6A–D, NAMPT protein expression was increased ~9 and 6 times in IL-1β and EC treated groups relative to controls. Similarly, ACT1 protein expression was increased by ~6 and 5.5-fold by IL-1β and EC treatments, respectively. Notably, SOD2 protein expression was similar between EC and IL-1β as it increased by 6.5 and 6.3 by IL-1β and EC, respectively.

**Figure 7:**
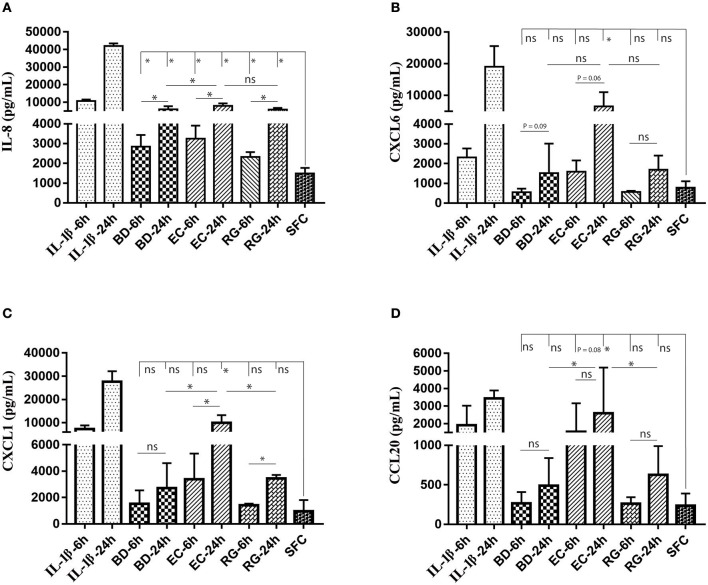
Changes in protein levels of selected cytokines in islets culture supernatant following incubation with bacteria and IL-1β. Selected cytokines included IL-8 **(A)**, CXCL6 **(B)**, CXCL1 **(C)**, and CCL20 **(D)**. Only IL-8 revealed significantly increased levels in all experimental treatments at both 6 and 24 h. Data are shown as averages ± standard deviations (*n* ≥ 3 donors) and are representative of three independent experiments. *P < 0.05; ns, not significant.

ELISA measurements of islet supernatants for IL-8, CXCL-6, CXCL-1, and CCL20 levels revealed time dependent increases by most treatments. These factors are known to increase during islet inflammation and diabetes (26, 27). Such increases were most pronounced by IL-1β (Figures 7A–D). However, only IL-8 was consistently increased by all bacteria at both 6 and 24 h (Figure 7A).

## Discussion

In the present study, human pancreatic islet responses to selected bacteria overrepresented in the intestine of T1D subjects were measured and compared *in vitro*. *Ruminococcus gnavus* (RG), a Gram-positive bacterium and a pathobiont-like species (i.e., member of gut commensals that have the capacity to behave as pathogens), showed a spike in relative abundance in T1D-susceptible children concomitant with decreased diversity of gut microbiome (15) was investigated in our study. *Bacteroides dorei* (BD), a Gram-negative bacterium significantly elevated in children becoming autoantibody positive (seroconverters) compared to controls (non-converters) prior to the appearance of the first islet autoantibody (14), was also included in our analysis. *Escherichia coli* (EC) is another Gram-negative bacterium but its abundance is not affected by gut dysbiosis. Since the latter bacterium is known to elicit a potent immunostimulatory effect, indicated by the release of proinflamatory cytokines from human mononuclear cells exposed to its LPS (20), EC was used to assess islet responses associated with BD and RG. IL-1β, a potent inflammatory cytokine associated with islet failure in both types of human diabetes (28, 29), served as a positive control.

Our RNA-Seq analysis of human islets exposed to selected gut bacteria revealed alterations in gene expression levels at both 6 and 24 h, with dominance in a number of DEGs by EC (459 and 310, respectively) followed by RG (305 and 225, respectively) and BD (157 and 151, respectively). It is noteworthy that the number of DEGs shared by each bacteria with IL-1β followed the same pattern. Although the reasons for such variation are largely unknown, the distinct structural features among bacteria probably account for these differences. Variation in the immunogenicity of LPS between EC and BD was recently reported (20). In line with our findings, higher levels of nuclear factor kappa B (NF-κB) induced cytokines e.g., IL-1β and IL-6 were observed in human mononuclear cells following incubation with LPS from EC compared to BD. Surprisingly, the increased production of the above mentioned cytokines by EC was associated with lower rate of diabetes incidence (20). Intraperitoneal (i.p.) injection of LPS from EC delayed the onset of T1D in non-obese diabetic (NOD) mice, whereas BD LPS had no effect (20). Moreover, splenocytes isolated from NOD mice 24 h following i.p. injection of EC LPS were less responsive to further *in vitro* stimulation by bacterial endotoxins compared to BD LPS (20). This suggests that continuous exposure to EC LPS, but not BD LPS, contribute to prevention of autoimmunity via increasing the host microbial tolerance. The distinct islet antibacterial response to RG is likely due to cell wall constituents such as peptidoglycans and lipoteichoic acid (30). Although the mechanism is still unclear, peptidoglycans and lipoteichoic acid are able to induce an inflammatory response comparable to that induced by LPS via activation of host pattern recognition receptors (31). This probably explains the shared features seen between both gram positive (RG) and gram negative (BD, EC) in the present study.

### Unique BD-Mediated Effects on the Human Islet Transcriptome

BD exposure led to unique changes in islet gene expression. *ADM2, ASNS*, and *CHAC1* were among the set of genes preferentially upregulated by BD. *ADM2* encodes adrenomedullin 2, also called intermedin, a novel secreted peptide belonging to calcitonin family hormones (32), with its expression being upregulated in different stress conditions including endoplasmic reticulum (er) and oxidative stress (33, 34). Interestingly, overexpression of *ADM2* in mice improved insulin sensitivity in adipocytes (35), suggesting its role in metabolism. The upregulated expression of *ASNS* by BD, which encodes asparagine synthetase, is known to increase the production of asparagine (ASN). ASN contributes to insulin secretion from pancreatic islets via accelerating the rate of cytosolic NADPH generation (36), demonstrating a metabolic role for *ASNS*. Interestingly, the released ASN is also sensed by bacteria and modulates the expression of nearly 17% of its genes increasing the rate of bacterial growth (37). This suggests a role for ASN in mediating the symbiosis between islets and bacteria. The exclusive activation of the tRNA charging/loading pathway by BD exposure confirms its ability to affect amino acid metabolism and protein synthesis. tRNA charging/loading with amino acids is mediated by the enzyme aminoacyl-tRNA synthetase that precedes mRNA translation by ribosomes (38). *CHAC1* encodes glutathione-specific gamma-glutamylcyclotransferase 1 that plays an important role in glutathione metabolism and is associated with glutathione depletion in stressed human islets (39). Glutathione contributes to balancing the redox status of the islets and its deficiency predisposes to diabetes (40–42). Additionally, *CHAC1* expression leads to increased inflammation during bacterial infection (43). *CHAC1* inhibition reduced il-6, il-8, and CCL2 secretion in cells stimulated with LPS or flagellin (43), suggesting a role for *CHAC1* in mediating tissue inflammation through controlling the release of cytokines. Its upregulation would clearly be proinflammatory, create insulitis and alter islet redox state. Genes encoding TGF-β3 and SMAD9 were downregulated by BD. TGF-β signaling regulates development, differentiation, and function of pancreatic β-cells (44, 45). TGF-β3 has been also suggested to play both immunomodulatory and anti-inflammatory roles (46, 47). Interestingly, SMAD9 downregulation was evident in transcriptomic profiling of isolated pancreatic islets from T1D patients (48). In conclusion, our results suggest that BD exposure uniquely affects insulin secretion, islet metabolic functions and islet cell differentiation while promoting inflammation.

### Unique RG-Mediated Effects on the Human Islet Transcriptome

RG exposure also resulted in unique gene expression changes. *BBC3* (BCL2 binding component 3) and *ATF3* (activating transcription factor 3) are two genes exclusively induced by RG in human islets. Both BBC3 and ATF3 were reported to play important roles in stress-induced β-cell apoptosis (49–51). Interestingly, *ATF3* upregulation was also found to mediate protection against Gram-positive bacteria by enhancing cytokine production during bacterial infection (52). In addition to that, *HAMP* (a gene coding for hepcidin) was listed among genes with increased expression in RG-treated islets. Hepcidin is well known for its role in iron metabolism (53). It has a known connection to the antimicrobial response as its concentrations are increased following exposure to bacteria (54). HAMP acts to starve bacteria of iron. Given that hepcidin expression in the pancreas is exclusive to insulin granules of islet β-cells (55), our analysis points to hepcidin as a critical antimicrobial peptide upregulated during insulin secretion. Iron also regulates different aspects of islet β cell functions including glucose stimulated insulin secretion (56). Wnt signaling plays a role in immunomodulation (57). The Wnt family member *WNT16* is exclusively downregulated in islets by RG. Interestingly, *WNT16* is downregulated in several autoimmune diseases including lupus and arthritis (58). Although earlier reports suggested abundant expression in the pancreas (59), the specific cells expressing WNT16 in the pancreas remains undefined. Notably, glucocorticoids are known for their immunosuppressive properties and a dose-dependent inhibition of *WNT16* was reported in mouse bone tissues following glucocorticoid treatment (60). Interestingly, overexpression of *WNT16* in mouse bone cells was able to revert glucocorticoid-induced damage (60). Taken together, these data point to WNT16 as an islet immunomodulator whose expression is downregulated under stress conditions.

### Bacteria-Specific Effects on the Human Islet Transcriptome

BD, EC, and RG treated islets shared some features. From the commonly upregulated list of cytokines/chemokines, three factors revealed the highest increase in expression levels in response to bacteria, especially RG, compared to IL-1β. These factors are *IL-23A, CCL3*, and *CCL4* (Table 2). A similar induction of IL-23A, CCL3, and CCL4 by bacteria in other cell types was reported (61–63), suggesting a major role for these cytokines in antibacterial defense. The upregulated transcript levels for IL-23A and CCL3 were found associated with high incidence of autoimmunity (64). Conversely, the role of CCL4 during T1D appears to be protective (65).

### Transcriptome Changes Shared by All Treatments

Five genes were found to be commonly downregulated by all bacteria and IL-1β exposure; *OXGR1, PRSS2, MS4A6A, PLCE1*, and *EPHA4*. *EPHA4* (EPH receptor A4) belongs to the protein-tyrosine kinase family. Dysregulated EPH/ephrin signaling is known to affect hormone secretion in human and mouse islets (66, 67). Although the biological relevance of the other downregulated genes, *OXGR1, PRSS2, MS4A6A, PLCE1*, to T1D is unknown, these factors might also have important roles in the pancreas. For instance, *Oxgr1* knockout mice revealed inflammatory changes in the middle ear with a significant degree of hearing loss, suggesting a role for OXGR1 in counteracting inflammation (68).

Our comparison analysis using IPA software revealed 47 genes upregulated by all bacteria and IL-1β. IL-1β transcription is strongly induced by all the bacteria providing a basis for these commonalities. These set of genes is dominated by cytokines (20/47), enzymes (7/47), and receptors (5/47). IL-1β expression is known to activate NF-kB pathway (69, 70), the increased expression of transcripts for RelB and NF-kB2 (P52) subunits of NF-kB observed in our RNA-Seq is most likely induced by islet overexpression of IL-1β. Such activation of the NF-kB pathway can have beneficial effects on cells (71). However, persistent release of NF-kB dependent factors, including inflammatory cytokines (e.g., IL-8, CXCL6, CXCL1, and CCL20), adhesion molecules (e.g., ICAM1), G protein coupled receptors (e.g., FFAR2 or GPR43), enzymes (e.g., PTGS2 and TRAFIP3, or A20), and matrix metallopeptidases (e.g., MMP1), could have very deleterious effects on islets (72–74). IL-1R signaling plays an important role during both diabetes progression and rejection of islet grafts during transplantation by modulating IL-1β action (75, 76). IRAK2, an enzyme that regulates IL-1β action (77), was also found to be increased in our RNA-Seq analysis by all groups. Such IRAK2 upregulation is probably associated with sustained auto-stimulatory effects of IL-1β on islet cells (71). It is noteworthy that, activation of NF-kB pathway was also accompanied with NF-kBIA (NF-kB inhibitor α) upregulation that was probably due to its continuous nucleocytoplasmic shuttling to counteract the islet damage induced by NF-kB (78). The release of four chemokines, namely IL-8, CXCL6, CXCL1, and CCL20, known to be regulated by NF-kB activation was partly confirmed using ELISA.

Our RNA-Seq analysis surprisingly revealed increased expression of both *IL-7* and *IL-7R* by all treatments. IL-7 is a cytokine known to be expressed by stromal cells in lymphoid tissues and bone marrow (79), but has never been reported in islets. The cellular source of these factors in human islets requires investigation.

It is noteworthy that *SLC6A14* (Solute Carrier Family 6 Member 14) which encodes an l-arginine transporter protein, was upregulated by all treatments. Islet exposure to L-arginine is known to increase insulin secretion (80) again suggesting bacterial influences on islet function. Exposure of airway epithelia to bacterial flagellin led to upregulation of SLC6A14 expression and increased uptake of L-arginine by the cells (81). Moreover, inhibition of SLC6A14-dependent L-arginine transport enhances bacterial attachment, suggesting an additional role for SLC6A14 in islets via inhibiting bacterial pathogenesis (81).

Upregulated expression of intracellular NAMPT, TRAF3IP2, and SOD2 was confirmed at the protein level using western blotting (Figures 5, 6). NAMPT encodes nicotinamide phosphoribosyltransferase, a rate-limiting enzyme for NAD(+) biosynthesis (82). NAMPT expression has been reported in islet β-cells (83). Interestingly, NAMPT upregulation through exogenous administration of nicotinamide mononucleotide corrected islet damage induced by inflammatory cytokines IL-1β and TNF-α (84), suggesting a protective role for NAMPT in during islet stress. TRAF3IP2 is implicated in NF-kB activation in many tissues during inflammation (85–87). SOD2 was abundantly expressed in islets, as indicated by its signal intensity in our immunoblots. Heterozygous deletion of SOD2 has been shown to cause impaired insulin secretion in a mice model of obesity (88).

### Signaling Pathways Activated by Islets Exposed to Bacteria

Our IPA core analysis confirmed the inflammatory nature of the islet antibacterial response. In line with increased *IL-1*β mRNA in our RNA-Seq DEGs, IL-1 signaling was found among the top canonical pathways upregulated by all treatments. Another upregulated pathway is the intracellular accumulation of nitric oxide synthase (NOS) which is associated with mitochondrial dysfunction and correlates with the severity of islet inflammation (89). The inflammatory response pathway was the top disease category altered by bacteria and cytokine treatment in our IPA functional analysis. The comparatively limited inflammatory response induced by BD complements our knowledge about its weak immunostimulatory effects (20). TREM1 (triggering receptor on myeloid cells 1) is the topmost activated canonical pathway in our system. Upregulation of TREM1 signaling pathway is associated with increased production of inflammatory cytokines and possibly contributes to islet damage (90–92). TREM1 upregulation was also noted to correlate with the severity of bacterial infection via enhancing NF-κB pathway (93, 94).

The limitations of the present study should be acknowledged. First, due to cost considerations, only eight human subjects were tested. This might affect the reproducibility of data when larger numbers of subjects are tested. Second, inclusion of commensal gut species not associated with islet inflammation were not evaluated. Thus, with the current design it is unclear whether the reported responses were specific to the tested bacteria or reflect a shared islet response to all bacteria. Third, other *in vivo* factors contributing to complexity of islet response e.g., genetic susceptibility and signaling provided by islet juxtaposed tissues are missing. Lastly, commercial bacterial strains from the ATCC were used in this study. Recent reports have demonstrated genomic variation and strain-specific functional adaptation of gut bacteria in individuals with diseases (95, 96). Therefore, bacteria isolated from diabetic individuals could induce a different islet response.

**Figure 8:**
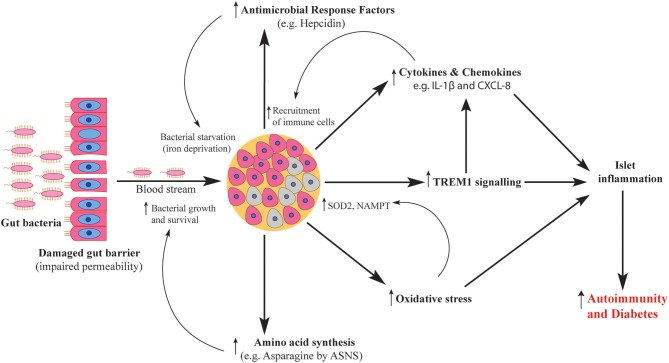
Schematic representation of human islet response against T1D-associated bacteria. Gut bacteria traverse the damaged gut barrier and reach the pancreas. Upon reaching the pancreatic islets, bacteria elicit islet response via several pathways including activation of antimicrobial response factors, increased production of cytokines and chemokines, activation of TREM1 signaling, increased oxidative stress levels and enhanced amino acid synthesis. The activated pathways contribute to both islet damage and microbial survival which ultimately cause islet inflammation and increase the severity of autoimmunity.

In summary, our *in vitro* system uncovered key features of the human islet response against invading gut bacteria and their products (Figure 8). BD and RG uniquely influenced the human islet transcriptome. These alterations would clearly affect islet function and survival. Although direct role of bacteria in T1D is under investigation, in the present study, proinflammatory features such as cytokine and chemokine induction fit with the observation of infiltrating immune cells into islets in human T1D. Future mechanistic studies involving *in vivo* models are needed to link these changes to T1D pathogenesis.

## Data Availability Statement

The datasets generated for this study can be found in GEO under the accession number GSE131320, https://www.ncbi.nlm.nih.gov/geo/query/acc.cgi?acc=GSE131320. GEO Accession viewer NCBI's Gene Expression Omnibus (GEO) is a public archive and resource for gene expression data.

## Author Contributions

AA and HJ: experimental design and execution, data analysis, and manuscript preparation. RH and NS: experimental design, data analysis, and reviewed the manuscript.

### Conflict of Interest

The authors declare that the research was conducted in the absence of any commercial or financial relationships that could be construed as a potential conflict of interest.
