# Supplementary material for: Human Islet Response to Selected Type 1 Diabetes-Associated Bacteria: A Transcriptome-Based Study
Source: Front Immunol. 2019 Nov 8;10:2623. doi: 10.3389/fimmu.2019.02623 (PMC6857727; doi:10.3389/fimmu.2019.02623)
Supplement: Supplementary file 1 [file Table_1.DOCX]

**Supplementary table 1. Bacterial strains used in the present study:**

| **Bacterial Strain** | **Reference no.** | **Type** | **Growth** | **Culture media** | **Accession no.** | **No. of scaffolds** | **genome size** | **consensus size** | **No. of reads per sample** | **Aligned reads** | **% mapped** | **% similarity in alignment** |
| --- | --- | --- | --- | --- | --- | --- | --- | --- | --- | --- | --- | --- |
| ***Bacteroides dorei***  **17883** | DSM 17855 | Gram negative | Anaerobic | Trypic soy broth | NZ_DS995528,-  NZ_D5995567 | 40 | 5,566,217 | 5,566,219 | 7,475,304 | 7,436,794 | 99.5 | 98.5 |
| ***Escherichia coli***  **DH5α** | Invitrogen  18258-012 | Gram negative | Aerobic | LB broth | NZ_JRYM010000 01-  NZ_JRYM010000 89 | 89 | 4,507,030 | 4,507,061 | 7,246,164 | 7,209,528 | 99.5 | 99.8 |
| ***Ruminococcus***  ***gnavus***  ***VPI C7-9*** | ATCC 29149 | Gram positive | Anaerobic | Trypic soy broth | AAYG02000001-  AAYG02000043 | 43 | 3,501,911 | 3,501,928 | 10124376 | 10055739 | 99.3 | 99.9 |
