# Supplementary material for: Human Islet Response to Selected Type 1 Diabetes-Associated Bacteria: A Transcriptome-Based Study
Source: Front Immunol. 2019 Nov 8;10:2623. doi: 10.3389/fimmu.2019.02623 (PMC6857727; doi:10.3389/fimmu.2019.02623)
Supplement: Supplementary file 2 [file Table_2.DOCX]

**Supplementary table 2. Human islet donors’ characteristics:**

| **Subject no.** | **Prodo ID** | **Age (y)** | **Ethnicity** | **BMI (kg/m2)** | **HbA1c** | **Diabetes?** | **Islet purity (%)** | **Islet viability** | **Cause of death** |
| --- | --- | --- | --- | --- | --- | --- | --- | --- | --- |
| 1 | HP-15324-01 | 58 | Caucasian | 19 | 4.9% | No | 95 | 95 | Stroke |
| 2 | HP-15338-01 | 41 | Black | 24.5 | N/A | No | 90 | 95 | Stroke |
| 3 | HP-16030-01 | 41 | Caucasian | 24.1 | 5.6% | No | 90 | 95 | Head trauma |
| 4 | HP-16098-01 | 36 | Hispanic | 23.5 | 5% | No | 90 | 95 | Stroke |
| 5 | HP-16125-01 | 21 | Caucasian | 22.8 | 5.2% | No | 85-90 | 95 | Head trauma |
| 6 | HP-16141-01 | 37 | Caucasian | 23.3 | 5.4% | No | 90 | 95 | Stroke |
| 7 | HP-16267-01 | 46 | White | 26 | 5.4% | No | 90 | 95 | Natural causes |
| 8 | HP-16315-01 | 49 | White | 26.5 | 5.2% | No | 90 | 95 | Head trauma |
